# A salutogenic approach to early labor midwifery care in Denmark, Sweden, and Switzerland: A qualitative study

**DOI:** 10.18332/ejm/206921

**Published:** 2025-07-10

**Authors:** Mie Gaarskjaer de Wolff, Susanne Grylka-Baeschlin, Antonia Ν. Mueller, Maja Bodin, Nanna Β. Pagh, Karin Ängeby

**Affiliations:** 1 Department of Obstetrics and Gynocology, Copenhagen University Hospital, Hvidovre, Denmark; 2 Research Institute of Midwifery and Reproductive Health, School of Health Sciences, ZHAW Zürich University of Applied Sciences, Winterthur, Switzerland; 3 Division of Midwifery, Bern University of Applied Sciences, Bern, Switzerland; 4 National Centre for Knowledge on Men's Violence Against Women, Uppsala University, Uppsala, Sweden; 5 Centre for Clinical Research and Education, Region Värmland, Karlstad, Sweden; 6 Department of Health Sciences, Faculty of Health, Science and Technology, Karlstad University

**Keywords:** qualitative research, midwifery care, maternity care, salutogenesis, latent phase, cross-national study

## Abstract

**INTRODUCTION:**

Early labor is critical in shaping birth experiences, and women seek midwifery care to manage challenges during this stage. A salutogenic attitude, focusing on positive experiences and health promotion, is needed instead of the current pathogenic maternity model. This study aimed to understand how midwives support women’s positive early labor experience and to identify key elements contributing to salutogenic midwifery care practices during early labor.

**METHODS:**

We employed a qualitative study design, conducting nine focus group discussions (FGDs) with 39 women in Denmark, Sweden, and Switzerland. First, data were analyzed thematically using an inductive approach to capture codes. Second, a salutogenic best-fit framework guided the analysis, structuring the data into three domains: comprehensibility, manageability, and meaningfulness. Within domains, sub-themes were identified to elucidate how midwifery care might contribute to positive early labor experiences.

**RESULTS:**

Comprehensibility was achieved when women trusted midwives to provide accessible, high-quality care tailored to individual needs to help them comprehend the maternity system. Manageability was experienced when midwives guided women through the uncertainties of early labor and provided plans when needed. Meaningfulness was created through availability, accessibility, and emotional support. Continuity of care was an underlying foundation for a positive experience of all three domains.

**CONCLUSIONS:**

A salutogenic midwifery approach, centering on flexibility and individualization, was essential to women’s positive experiences cross-nationally. Salutogenic midwifery care during early labor reduced anxiety and enhanced emotional resilience. Our findings underscore the importance of salutogenic midwifery care for women in early labor, which should be recognized and implemented in maternity care settings.

## INTRODUCTION

Women’s early labor experiences are pivotal in shaping childbirth experiences^1^. This phase of labor, whose length can vary significantly among women from a few hours to days^2,3^, is characterized by various symptoms, including contractions, labor pains, and mixed emotions, and presents a unique set of challenges^4^. Many women and their partners actively seek midwifery support to manage the physical sensations and navigate the emotional complexities of early labour^5,6^. This support is not just a service but a beacon of reassurance and empowerment, contributing significantly to a positive childbirth experience^7,8^.

However, providing supporting early labor care is not possible without recognizing its complexities for individual women. In many birth institutions, a prevalent clinical practice is to delay hospital admission during this phase. This approach is primarily driven by a wish to prevent unnecessary interventions, such as augmentation or cesarean sections^9-13^. While this practice has its merits, it may also lead to unintended consequences, such as increased anxiety or feelings of neglect among women who need professional support but are advised to remain at home^5,8,14^.

The relationship between midwifery care practices and birth outcomes is not fully understood and no causal link between delayed admission and the reduction of unnecessary interventions has been established^2^. This uncertainty underscores the need for a nuanced understanding of how care during early labor can be optimized. The World Health Organization (WHO) advocates for personalized early labor care, emphasizing that interventions should be tailored to each woman’s needs and preferences instead of a policy of delaying labor ward admission^15^.

Whereas maternity care systems and approaches differ across European maternity care settings^16^, comparing women’s early labor care experiences across these countries can help uncover commonalities pointing to fundamental aspects of supportive care during early labor. Simultaneously, this approach has the potential to highlight how specific care elements are shaped by cultural, organizational, and policy environments^17^. By capturing these nuances, a more comprehensive understanding can be achieved of how early labor midwifery care should be tailored across varying healthcare settings, ultimately informing practices that promote positive childbirth experiences globally.

In recent years, a scientific and clinical agenda focusing on high-quality maternity and salutogenic midwifery care practices has gained traction^18,19^. The salutogenesis theory of Antonovsky^20^ has contributed to understanding of how health is created and maintained. Systematic reviews have repeatedly suggested that a salutogenic approach to midwifery care may enhance outcomes such as maternal satisfaction, including women feeling in control and experiencing comfort^19^.

Therefore, this study aimed to understand how midwives support women’s positive early labor experience and to identify key elements contributing to salutogenic midwifery care practices during early labor. The research questions were: 1) ‘What constitutes good midwifery care during early labor?’; and 2) ‘Which factors are described by women as salutogenic early labor midwifery care practices in Denmark, Sweden, and Switzerland?’.

## METHODS

### Study design and setting

We conducted a qualitative study using data from nine focus group discussions (FGDs) in three European countries [Denmark (DK), Sweden (SWE) and Switzerland (CH)]. Data were collected between August 2021 and November 2023. The study was embedded within three more extensive national research studies and was conceptualized by the authors. Participants from Denmark gave birth in one urban tertiary hospital, while those from Sweden originated from a national sample and gave birth in four different hospitals. Swiss women gave birth in various hospitals and birth centers in the German part of the country. Table 1 provides an overview of the maternity care systems across the three settings, including access to early labor care.

**Table 1:** Characteristics of maternity care settings in Denmark, Sweden, and Switzerland

Denmark	Sweden	Switzerland
**Maternity care organization**	Tax-funded universal maternity care. Shared care between midwives and general practitioners and involvement of obstetricians in case of pregnancy or labor complications^35^. In the study setting, eight participants had access to a newly implemented midwife-led continuity of care model^36^.	Tax-funded, free maternal and child health care system. midwives are primary caregivers. In case of complications, the obstetrician takes responsibility and supports the midwife as appropriate. Options such as continuity of midwife caregiver, home birth and birth center care are not traditionally offered by the health services, only in small research projects^37^.	Primarily obstetrician-led, some midwife-led cases. Insurance covered^38^. In the study setting, four participants had access to a continuity of care model.
**Antenatal care (including antenatal birth preparation**	5–7 midwifery consultations with the same midwife 2–3 consultations with general practitioner (GP) 2 ultrasounds Antenatal birth preparation included in routine midwifery visits, webinars, and digital resources.	Midwife-led care: 8+ visits with the same midwife from GA 9 to postpartum, with obstetrician referral as needed^37^. Antenatal birth preparation Midwife-led: free of charge cohesive groups with multiple meetings, large group lectures, digital group meetings, and individual digital information.	7 visits, including 2 ultrasounds. Antenatal birth preparation Midwife-led: group and private classes, partially insurance-covered.
**Early labor care**	Women are encouraged to stay home during early labor, but if needed, the hospital has a dedicated early labor unit with four rooms, a shared midwifery team, and no one-to-one care.	Midwife-led: women are encouraged to stay home during early labor; care is provided if admitted to the labor ward. Some hospitals offer a stay at a patient hotel until established labor.	Variable care models: caseload midwives may provide early labor care at home and limited one-to-one care in hospitals.
**Labor care**	Midwife-led: one-to-one care policy in the labor ward.	Midwife-led: one-to-one care policy in the labor ward.	Limited one-to-one care, continuity, and one-to-one midwifery care are prioritized in midwife-led settings.
**Postpartum care**	1 to 2 nights’ stay in the maternity ward for nulliparous women. 4–6 hours in labor ward for multiparous women and telephone consultation and visit at department.	Midwife-led: immediate postpartum care; stay in the labor ward for 6+ hours or transfer to the postnatal ward if needed or go home after uncomplicated birth.	2 to 3 nights’ stay in maternity ward; insurance covers up to 10–16 midwife visits post-birth for primiparous or at-risk mothers.

### Sampling strategy and participants

We sampled participants with descriptions of midwifery care during early labor among women with a spontaneous onset of labor at term. Danish participants were recruited from a study using data from surveys and medical records about the characteristics of early labor. Swedish participants were recruited from a cohort of primiparous women who participated in an RCT to study whether a mobile app could reduce early labor distress. Women willing to participate in a follow-up interview after childbirth and identifying having an early labor at home >18 h, were invited to participate. Swiss participants participated in developing a tool to improve early labor care^21,22^ and were recruited through independent midwives who provided postpartum care. In all countries women were approached by the authors and given verbal and written study information about participating in an FGD and gave informed consent when participating about experiences of midwifery care during early labor.

### Interview guide and interview procedure

All authors contributed to the development of the shared interview guide designed to explore women’s experiences of midwifery care in early labor. The FGDs followed a structured guide to explore women’s experiences of midwifery care during early labor. The interviews were designed to encourage open dialogue among participants, focusing on personal experiences and reflections without aiming for consensus. The interviews covered key domains: the onset of labor, time spent at home, contact with the labor ward, and, through all interactions, the experiences with midwifery care both over the phone and at the hospital. Sub-questions probed specific domain aspects, such as symptoms, emotional experiences, the decision-making process for contacting the hospital and hospital admission, and examples of supportive care. The nine FGDs were facilitated by the authors who are all midwives (MW, SG-B, MB, AM, NP, KÄ). At the interviews were two midwives, one of the authors was responsible for the overall facilitation and the other author was responsible for writing notes and keeping track that all participants having the chance to contribute to the conversation. The FGDs in Sweden and Denmark were held online (n=5). The Swiss FGDs were held either face-to-face (n=3) or on-line (n=1) according to the availability and wishes of participants and COVID-19 restrictions. All FGDs were recorded (audio and or video) and transcribed.

### Ethical considerations

The study followed the ethical principles of the Helsinki Declaration for Medical Research^23^. Participants gave signed informed consent. The study was approved by the Research Ethical Board of the Capital Region, Denmark (Ref. nr: P-2022-261), The Swedish Ethical Board (ref.no 202103028) and the Ethics Committee of Zurich, Switzerland (BASEC-Nr. 2021-00687).

### Data analysis

To define the salutogenic aspects of midwifery care practices during early labor, we applied the best-fit framework synthesis of salutogenic domains comprehensibility, manageability, and meaningfulness as described by Mathias et al.^24^. Data were analyzed thematically, following the phases outlined by Braun and Clarke^25^. The coding process was initially inductive, directed by the two research questions, and later deductive when the salutogenic framework was used to organize codes and themes.

In the first phase, researchers from each country read their country-specific transcripts to familiarize themselves with the data and started coding using NVivo^26^ or Atlas^27^. All authors then discussed codes (translated to English) and potential themes. In the next phase, the digital visual tool Miro Board^28^ was used to organize the work with codes from all interviews and sort them according to the salutogenic domains. This process led to the identification of sub-themes, which unfolded in the three domains^24^.

## RESULTS

### Findings

A total of 39 women participated in the study’s nine FGDs [online (n=6), face-to-face (n=3)]. The median time from birth to FGD was 22 (8–41) weeks. The mean age was 32.5 years (SD=1.5). In total, 93% (n=36) had a vaginal birth. Most women were primiparous (n=31), but the Danish FGDs included multiparous participants (n=8). Most participants gave birth at public hospitals (n=36; 92%) (Table 2). In Denmark eight of the participants were in a continuity of care model and were cared for by the same midwife during antenatal and intrapartum care. In Switzerland (n=4) had access to continuity of care in Sweden none of the participants had access to continuity of care models. A total sample of 30% of participating women were cared by a known midwife.

**Table 2:** Characteristics of the interviews and the participants in Denmark, Sweden, and Switzerland

*Characteristics*	*Denmark*			*Sweden*		*Switzerland*			
	*FGD 1*	*FGD 2*	*FGD 3*	*FGD 1*	*FGD 2*	*FGD 1*	*FGD 2*	*FGD 3*	*FGD 4*
**Number of participants**	6	4	5	2	4	5	4	4	5
**Type of interview**	online	online	online	online	online	face-to-face	face-to-face	face-to-face	online
**Participation** (weeks after birth)	32	17	28	32	30	17	18	18	25
**Age** (years), mean (range)	33 (27–39)	30 (23–34)	33 (29–36)	35 (34–36)	33 (29–36)	31 (29–34)	32 (29–34)	33 (29–34)	34 (29–41)
**Parity**, n (%) Primiparous Multiparous	3 (50) 3 (50)	1 (25) 3 (75)	3 (60) 2 (40)	2 (100) 0 (0)	4 (100) 0 (0)	5 (100) 0 (0)	4 (100) 0 (0)	4 (100) 0 (0)	5 (100) 0 (0)
**Cohabitation**, n (%) Married/cohabitant Single	6 (100) 0 (0)	4 (100) 0 (0)	5 (100) 0 (0)	2 (100) 0 (0)	4 (100) 0 (0)	4 (80) 1 (20)	4 (100) 0 (0)	4 (100) 0 (0)	5 (100) 0 (0)
**Gestational age at birth** (weeks) range	39–42	38–41	40–42	39–40	39–41	41–42	39–42	38–42	37–42
**Mode of birth**, n (%) Spontaneous Instrumental Cesarean section	5 (83) 0 (0) 1 (17)	4 (100) 0 (0) 0 (0)	4 (80) 1 (20) 0 (0)	2 (100)	4 (100)	5 (100) 0 0	3 (75) 1 (25) 0 (0)	3 (75) 1 (25) 0 (0)	3 (60) 0 (0) 2 (40)
**Place of birth**, n (%) Public hospital Private hospital Birth center	6 (100) 0 (0) 0 (0	4 (100) 0 (0) 0 (0)	5 (100) 0 (0) 0 (0)	2 (100) 0 (0) 0 (0)	4 (100) 0 (0) 0 (0)	3 (60) 0 (0) 2 (40)	3 (75) 1 (25) 0 (0)	4 (100) 0 (0) 0 (0)	5 (100) 0 (0) 0 (0)

FGD: focus group discussion.

### Thematic findings

The thematic data analysis was structured by the three domains of salutogenic midwifery care: comprehensibility, manageability, and meaningfulness^24^. The identified subthemes unfold under each domain. Continuity of care was identified as a theme underpinning the experiences across domains (Table 3).

**Table 3:** Salutogenic domains and secondary themes

*Comprehensibility*	*Manageability*	*Meaningfulness*
Conformity between individual needs and midwifery care	Finding inner strength	Building relationships fosters feelings of safety and security
Birth preparations – bridging expectations and reality	Clear guidance	Being confirmed in normality as an individual
Staying close to midwifery care	Involvement of the partner	
Continuity of care	Continuity of care	Continuity of care

### Comprehensibility

Women described how personalized care, clear guidance, and accessible support helped them process the uncertainties of early labor and feel empowered throughout this phase.

*Conformity between individual needs and midwifery care*

Women consistently highlighted the importance of midwives tailoring care to meet individual needs and trusted midwives to help them transition from home to the hospital. Women valued when midwives focused on their specific circumstances rather than strictly adhering to clinical protocols, enabling them to decide when to seek hospital admission.

No single approach worked for everyone. Some women found safety and comfort at home, while others preferred the hospital. They appreciated midwives who acknowledged these differences and adapted accordingly. However, when midwives implicitly promoted staying at home as the ideal, some women felt this expectation pressured them to endure longer at home, which led to feelings of inadequacy or failure:

*‘It felt like it was up to me to stay home as long as I could manage. I think it becomes difficult when you say ‘manage’ because it’s a bit like the “good girl” syndrome or something, where you feel like, as you've heard, so many people go in only to be sent back home. So, I think I really pushed it too far.’* (FGD 1, SWE)

*Birth preparations - bridging expectations and reality*

Antenatal education was critical in preparing women cognitively for early labor. Differences in antenatal preparation across the three settings were notable (Table 1). While the Danish setting offered limited in-person antenatal birth preparation, the Swedish and Swiss contexts provided more antenatal education integrated in standard maternity care. Hence, Danish women expressed dissatisfaction, particularly with their reliance on private classes:

*‘When it comes to childbirth preparation, I have quite strong opinions about it, I must say ... And then many can't afford it, and it’s not cheap such a course. And we just said, “Okay, we'll do it”, but many can't. And I think they shouldn't be faced with that huge disadvantage going into childbirth unprepared.’* (FGD 1, DK)

Women sought accurate and nuanced antenatal information about early labor and highlighted that generic advice often failed to reflect the complexity of early labor trajectories. This standardized approach left women unprepared when their experiences did not align with these narratives:

*‘I feel we've looked at it wrong [early labor]. We were told that it could be shorter or longer and that the best thing to do in this phase is to try to relax a little, withdraw as much as possible, and maybe watch a film or take another bath.’* (FGD 4, CH)

Uncertainty about early labor symptoms contributed to feelings of confusion and frustration. Many women described feeling unprepared for the variability of symptoms, such as irregular contractions or prolonged latent phases. A lack of alignment between expectations and the lived experience of early labor often undermined confidence and added to the mental burden. Several women entered labor with little to no expectations of what it might entail and, in hindsight, described it as the most challenging part of their birth experience:

*‘I would have liked the choices to be clearer. Either to have been prepared in advance, like if things don't progress, this is roughly how long it's reasonable to stay at home without assistance, or this is roughly how long it's reasonable to go without sleep before we assess that you need help. I was completely exhausted, in a lot of pain, and then I had to decide if I wanted to sleep here or there or somewhere else, and I found that a bit difficult’*. (FGD 1, SWE)

Midwifery care was central in bridging the gap between expectations and reality. For many women, preconceived notions about hospital care – such as the assumption that resources would be too strained, were proven wrong by supportive and attentive midwives.

*Staying close to midwifery care*

Women emphasized the importance of access to midwifery care during early labor, whether through telephone, access to hospital rooms, or the possibility of home visits. Proximity created a sense of safety and security in knowing that professional support was available:

*‘Then she told me just to call again. And knowing that I could come at any time was somehow a good feeling at that moment. Moreover, that was also the case when I arrived, when I already had such strong contractions that walking and standing were no longer so easy, yet it was such a welcoming feeling’*. (FGD 3, CH)

Women highlighted the importance of feeling supported at home. Dialogue during early labor made the home a safe and viable place to labor, especially when clear plans for follow-up were established. Women with access to home visits described this as a valuable opportunity to stay home while feeling secure with professional assistance.

### Manageability

Women valued tailored guidance, which encouraged them to embrace individual coping strategies. This support, particularly when paired with actionable plans and the practical involvement of partners, helped them to navigate the uncertainties of early labor with confidence and resilience.

*Finding inner strength*

Women described the importance of recognizing their capacity to manage early labor, particularly when antenatal preparations felt insufficient. Midwives helped women reframe self-doubt into confidence, encouraging them to trust their ability to handle labor demands:

*‘[I felt that] I must sleep. Otherwise, I won't be able to handle this once it (the active phase) starts if I don't get some sleep. Because then I was so tired that I felt completely exhausted. But then she [the midwife] said you should know you have so much more strength than you think. Even if you haven't slept, you will still have energy left for it.’* (FGD 1, SWE)

Empathetic communication and realistic guidance about the variability of early labor helped women feel empowered to adapt to unexpected circumstances. Encouragement and reassurance were crucial in assisting women to view their progress as natural and achievable rather than a reflection of failure:

*‘I was on the verge of tears. “What's wrong with me?”, “Why can't I give birth to this baby?” ... And then I remember that she hugged me and said, “There's absolutely nothing wrong with you; you’re just a first-time mother”. And just like that, that comment made a world of difference.’* (FGD 3, DK)

*Clear guidance*

The importance of having a clear plan for managing early labor was evident, co-created plans offered structure and reassurance. Receiving regular updates and actionable guidance reassured women and reduced feelings of anxiety and uncertainty. These plans were vital when labor did not progress as expected, providing women with safety and control.

Women emphasized the importance of midwives’ guidance during early labor, particularly in interpreting symptoms, understanding progress, and making informed decisions. Midwives helped women contextualize their experiences by piecing together physical sensations, emotional responses, and labor progression into a coherent whole:

*‘I couldn’t have thought of more myself. So, I was pleased that the midwife took over. At 8 o'clock, she called me and said, “Come on, we’ll do a check-up at the hospital at 9 o'clock”. And then everything was fine, and she gave me acupuncture and wrote me a plan of what I should do and when (laughs).’* (FGD 2, CH)

Women appreciated guidance in breathing techniques, relaxation strategies, and suggestions of birthing positions, which helped them feel supported and less overwhelmed. The midwife’s role extended beyond practical advice; their supportive presence and professional expertise created a sense of trust and capability:

*‘She [the midwife] entered the room with a completely different calmness. I think she noticed I had been breathing incorrectly. So, she came in and showed me how to breathe in a different, much slower, and much calmer way, and just that made everything feel much better immediately.’* (FGD 1, SWE)

*Involvement of the partner*

Partners were seen as vital sources of emotional and practical support, and women consistently highlighted the importance of the midwives’ involving partners or other support persons in the early labor process:

*‘She [the midwife] told him what he should do. And then, um, she gave him a chair by the bath, he could sit next to me, and she said, “sit there” and gave him a glass of water and said, “make sure she drinks a lot” and, um, instructed him a bit about what he could do.’* (FGD 3, CH)

Midwives who actively engaged with partners strengthened the support network for the laboring woman, reducing her burden and creating a sense of shared responsibility. By validating partners’ roles during early labor, midwives fostered collaboration and teamwork.

### Meaningfulness

Women described how midwifery care provided emotional stability, validated their unique experiences, and reassured them that their feelings and symptoms were normal. Midwives fostered meaningful connections and made women feel valued, supported, and secure during early labor. These relationships provided reassurance and allowed women to engage with the birthing process emotionally.

*Building relationships fosters feelings of safety and security*

Women expressed a strong desire to build relationships with midwives during early labor, even when this phase occurred at home. They appreciated midwives who took the time to listen, understand their narratives, and refer to documented birth plans. This attentiveness created a sense of connection and trust. Upon hospital admission, women appreciated the opportunity to form relationships with midwives. However, some felt these interactions could have been more supportive, particularly in providing information and engaging in dialogue about labor progression. A sense of safety and security was closely tied to midwives’ ability to convey trust, competence, and commitment to support each woman’s unique birth trajectory:

*‘We agreed that if no contractions came, I should come in after about three hours for a check. Otherwise, I should just call if they did start. And then they started slowly after about an hour and continued a bit, so I called again and said they had started, and I was actually feeling okay and wanted to stay home for now.’* (FGD 1, DK)

*Being confirmed in normality as an individual*

By verbalizing the women’s early labor symptoms and sensations, midwives helped women contextualize their experiences and feel reassured about their progress:

*‘She [the midwife] told me to describe what had happened and if I thought it was contractions, and she said, “Yes, that sounds like the start of labor”. And I thought that was good, that you don’t have to worry that it’s something that doesn’t belong.’* (FGD 3, CH)

Normalizing symptoms and emotional mirroring helped women feel confident and engaged in their birthing. Women emphasized the importance of being treated as unique individuals and valued midwives who adjusted care to their specific needs and acknowledged their vulnerability. A respectful and calm approach was experienced as emotionally supportive:

*‘That thing about being understood or being met. It’s a lot to expect that they [the midwives] can read us all. But maybe it’s because it’s such a vulnerable situation. That's why it means so much whether one feels understood or heard. Because it's very vulnerable, especially as a firsttime mother, when you don't know what you’re getting into.’* (FGD 2, DK)

Midwives who brought their clinical competence and personal understanding into encounters created a strong sense of respect and value, crucial for emotional support during early labor. Multiparous women consistently described how their previous birth experiences shaped their approach to managing early labor and provided insight into what to expect, what they wanted, and how to cope emotionally and practically.

### Continuity of care across domains

Continuity of care was identified as an essential aspect within all three salutogenic domains as a central concept contributing to women’s experiences of coherence, support, and connection. For ‘Comprehensibility’, continuity of care enhanced women’s understanding and navigation by providing consistency and familiarity. Women emphasized the value of being cared for by a midwife familiar with their personal history, preferences, and birth plans. This knowledge allowed for tailored guidance and facilitated comprehension of labor progression and decision-making, such as the timing of hospital admission:

*‘It helped me a lot that the midwife already knew me. Um, simply from the pregnancy check-up and because I knew her personally beforehand, and we talked a lot about what I wanted or couldn’t imagine.’* (FGD 2, CH)

For ‘Manageability’*,* women described the benefit of having direct access to a known midwife, which reduced barriers to seeking support and facilitated timely and tailored advice. The trust established during antenatal care enabled women to rely on their midwife for guidance, lessening the emotional and cognitive burden of navigating labor independently:

*‘I only had contact with my midwife, and she calmed me down because we could call her directly. We spoke with her a couple of times, and there were those five hours between the first and second time I called her. She was calm, and it was nice to talk to her. She wanted to hear how I was doing so I could call her when needed.’* (FGD 3, DK)

Women expressed that knowing that their midwife would be present during labor, alleviated anxiety about meeting unfamiliar caregivers, allowing them to focus on managing labor more effectively.

For ‘Meaningfulness’, continuity of care fostered meaningful connections between women and midwives, strengthening women’s emotional resilience during early labor. Women felt valued and supported by midwives who understood their needs and circumstances. For those with psychological sensitivities, continuity provided stability and emotional security. This connection added a layer of meaning to women’s experiences, turning a potentially isolating or challenging phase into one marked by reassurance and empowerment:

*‘I felt incredibly seen and heard, maybe because she, for example, she [the midwife] knew us by name. It's such a small thing but feeling seen as a human being ... it became more personal. Maybe that's also what made her able to create a connection between her and us, which made us feel more secure in it.’* (FGD 2, DK)

## DISCUSSION

The study was the first to explore early labor midwifery care from a salutogenic perspective, focusing on how midwives contributed to women’s experiences. The result showed that the experiences of women who gave birth in three different European countries and contexts covered all three domains of salutogenic care. ‘Comprehensibility’ was accomplished with personalized care and accessible support, ‘Manageability’ by valuable guidance from midwives, including individualized actionable plans to navigate early labor, and ‘Meaningfulness’ by confirming normality and providing security.

In addition, we found that continuity of care was central to all salutogenic dimensions outlined in the framework by Mathias et al.^5^. Knowing the midwife meant more straightforward access to information and care, as we found that they had less hesitancy in contacting the midwife. This finding aligns with results from a scoping review about women’s care needs, which found that women and their partners valued professional guidance, reassurance and preferred continuity of care models.

Also, reassurance and building trusting relationships with midwives is a significant need of couples in early labor as it has been described in previous research exploring what women value in midwife continuity of care models^5,29^. This study highlighted that women feel more secure and less anxious during early labor if they know the midwife. In its recent position paper, the WHO favored midwife-led continuity of care models^30^. However, in most studies investigating midwifery continuity of care models, it remains unclear how care was provided during early labor, leaving a need for further research to investigate the effects of midwife-led continuity of care during early labor^17^.

We found that access to midwifery care during early labor was essential for women’s positive experiences and allowed women to understand symptoms, cope with uncertainties, and feel emotionally connected. Salutogenic midwifery care provided reassurance and helped mirror women’s experiences, validating that their symptoms and sensations were part of a normal, albeit individual, labor trajectory. Being actively involved in deciding when and how to seek care was particularly important in shaping perceptions of midwifery care as accessible, creating a sense of autonomy and shared responsibility. Conversely, standardized care pathways were perceived as less accessible, leaving women feeling dismissed or unsupported, as described previously^1^. Care accessibility was pronounced for women who felt their labor progression deviated from what they had been told was ‘typical’, which is also highlighted in previous research^31^. We found that care was experienced as positive when midwives reframed symptoms, contextualized their experience and provided individual plans for continued labor work. Traditional organizational structures, however, often marginalize women’s care needs by delaying access to care during early labor. This approach is partly influenced by the need to manage limited resources and workloads and an assumption that delaying hospital admission helps avoid unnecessary obstetrical interventions^7,32^.

In contrast, the WHO advocates tailoring care to women’s needs and experiences without evidence to support delaying care^15^. Our findings confirmed previous findings^5^ that women experienced midwifery care that was provided with a personalized approach of widening access to care during early labor, positively impacted women’s birth experiences regardless system-level differences in midwifery models of care. However, policymakers need to restructure the approach from delaying care to tailoring care prospectively^33^.

Although this study focused on salient midwifery care experiences across country settings, we also found that some system-level differences were impacting women’s experiences. The participating women in Sweden and Switzerland are offered midwife-led antenatal education as part of the care during pregnancy, while this was not the case for women in the Danish setting, and Danish participants expressed significant dissatisfaction with antenatal birth preparation not being included in standard care. Another difference was the access to early labor care that was an option in the Danish setting but not in the other settings. The main findings that the salutogenic aspects of antenatal education, access to early labor care and continuity of care were important to women across all settings, needs further attention.

### Strengths and limitations

A strength of the present study is the use of the salutogenic framework, which made it possible to focus on strengthening salutogenic midwifery care practices instead of the often-described negative aspects^34^. The drawback of using a framework is that it might have hindered us from regarding other relevant issues in the dataset. However, the combined method with inductive coding as the first step and the deductive second step in the data analysis helped us acknowledge all the data. Another strength is cross-national data collecting, which allowed us to focus on similarities and identify differences, strengthening the results’ trustworthiness. Despite differences in the system-level proved midwifery care across the three settings, women’s experiences were overall similar in all settings. Mutual discussions in the author group helped us to discover and manage cultural differences and pre-understandings. Finally, it should be mentioned that the Danish setting included multiparous women, which made the samples slightly uneven. However, we believe that the experiences of multiparous women added critical perspectives and deepened our understanding of the phenomenon. As our focus was on descriptions of salutogenic midwifery care, adding the experiences of multiparous women who had more than one birthing experience added nuances and emphasized that the same aspects of care that were described as supporting positive experiences were the same across parity, strengthened the trustworthiness of the findings.

## CONCLUSIONS

Salutogenic midwifery care was essential for successful early labor care experiences across the three countries. Women had similar positive experiences, highlighting the importance of an individualized and easily accessible approach. Continuity of care models showed benefits, offering familiarity, reduced anxiety, and enhanced women’s emotional resilience during early labor. A salutogenic midwifery approach can be implemented in more traditional, medicalized settings, and the potential needs organizational recognition.

## Data Availability

The data supporting this research are available from the authors on reasonable request.
